# Induced endothelial cells from peripheral arterial disease patients and neonatal fibroblasts have comparable angiogenic properties

**DOI:** 10.1371/journal.pone.0255075

**Published:** 2021-08-10

**Authors:** Jack D. Hywood, Sara Sadeghipour, Zoe E. Clayton, Jun Yuan, Colleen Stubbs, Jack W. T. Wong, John P. Cooke, Sanjay Patel

**Affiliations:** 1 Heart Research Institute, Newtown, NSW, Australia; 2 Sydney Medical School, University of Sydney, Camperdown, NSW, Australia; 3 RNACore, Houston Methodist Research Institute, Houston, Texas, United States of America; 4 School of Life Sciences, Chinese University of Hong Kong, Hong Kong, China; 5 Department of Cardiovascular Sciences, Houston Methodist Research Institute, Houston, Texas, United States of America; 6 Department of Cardiology, Royal Prince Alfred Hospital, Camperdown, NSW, Australia; University of Edinburgh, UNITED KINGDOM

## Abstract

Induced endothelial cells (iECs) generated from neonatal fibroblasts via transdifferentiation have been shown to have pro-angiogenic properties and are a potential therapy for peripheral arterial disease (PAD). It is unknown if iECs can be generated from fibroblasts collected from PAD patients and whether these cells are pro-angiogenic. In this study fibroblasts were collected from four PAD patients undergoing carotid endarterectomies. These cells, and neonatal fibroblasts, were transdifferentiated into iECs using modified mRNA. Endothelial phenotype and pro-angiogenic cytokine secretion were investigated. NOD-SCID mice underwent surgery to induce hindlimb ischaemia in a murine model of PAD. Mice received intramuscular injections with either control vehicle, or 1 × 10^6^ neonatal-derived or 1 × 10^6^ patient-derived iECs. Recovery in perfusion to the affected limb was measured using laser Doppler scanning. Perfusion recovery was enhanced in mice treated with neonatal-derived iECs and in two of the three patient-derived iEC lines investigated *in vivo*. Patient-derived iECs can be successfully generated from PAD patients and for specific patients display comparable pro-angiogenic properties to neonatal-derived iECs.

## 1. Background

Peripheral arterial disease (PAD) is a significant contributor to global mortality and morbidity [1–4]. Revascularisation procedures are the gold standard therapy for severe disease [2, 5]. However, substantial subsets of patients are unsuitable for surgical or endovascular interventions due to pre-existing morbidity or an extremely calcified vasculature [6], and restenosis and/or thrombosis of vessels or bypass conduits is common after an intervention [7–10]. Therapeutic angiogenesis via cellular therapy, the stimulation and augmentation of vessel formation via the administration of exogenous cells [11], is a promising strategy for treating such patients [12].

Endothelial-like cells produced from induced pluripotent stem cells (iPSCs) have been demonstrated to be pro-angiogenic in pre-clinical models [13–18]. Recently, dermal fibroblasts have been transdifferentiated to endothelial-like cells, referred to as induced endothelial cells (iECs) [16, 19–22]. The potential benefits of direct transdifferentiation of somatic cells to iECs over reprogramming to full pluripotency to produce endothelial-like cells include potentially reducing production times and increased efficiency [16, 23–25]. Multiple studies have found that iECs possess pro-angiogenic properties, augmenting the restoration of blood flow in pre-clinical murine models of PAD [16, 19–21], with iECs shown to be equivalent to endothelial-like cells derived from iPSCs [16].

Recent results obtained for human iECs produced using lentiviral vector induced over-expression of transcription factors ETV2, FLI1, GATA2 and KLF4 used neonatal fibroblasts as the somatic cell type undergoing transdifferentiation [16]. However, the therapeutic promise of reprogrammed cells such as iECs lies in the capacity for autologous iECs to be produced directly from a patient’s somatic cells. Such patient-derived cells would theoretically have reduced immunogenicity upon transplantation back into the patient [24–26]. In addition, the use of lentiviral vectors in reprogramming, in particular producing iPSCs, has been demonstrated the cause genetic, epigenetic, and transcriptional abnormalities [27–30]. A non-integrating transdifferentiation strategy may be safer and have a simpler regulatory roadmap [16, 22]. Non-integrating factors, such as mmRNA, have been used successfully in reprogramming to pluripotency and transdifferentiation and offer a means of producing clinical grade cells [31–36]. In contrast, alternative techniques such as the use of small molecules alone in reprogramming is relatively inefficient [37, 38].

Given the above, it is yet been determined whether the pro-angiogenic effects previously shown in iECs can be replicated in iECs generated from patients with advanced atherosclerotic disease. Such patients are typically of advanced age and have an array of risk factors including smoking, diabetes, hypertension, hyperlipidaemia, among others. Determining whether patient-derived iECs are pro-angiogenic is an important step to determining whether these cells may be therapeutic in patients with cardiovascular disease. In addition, we wished to assess the feasibility of using modified messenger mRNA (mmRNA) encoding the transdifferentiation factors, rather than viral vectors.

Our objective was to perform a proof of concept study to determine whether dermal fibroblasts obtained from PAD patients could be transdifferentiated into iECs using mmRNA, and to establish whether they had comparable properties found in neonatal-derived iECs. We carried out assays to directly compare the *in vitro* characteristics of neonatal-derived and patient-derived iECs. The recovery of blood flow in a murine hindlimb ischaemia model of PAD was measured using laser Doppler to compare the *in vivo* pro-angiogenic capacity of the neonatal-derived and patient-derived iECs. Our results indicate that patient derived iECs possessed similar angiogenic properties to neonatal-derived iECs. These findings support the concept that iECs could be produced for therapeutic purposes from PAD and CAD patients.

## 2. Methods

### 2.1. Establishing patient derived cell cultures

Human dermal fibroblasts were isolated from 5mm full-thickness skin biopsies obtained from four peripheral arterial disease patients undergoing carotid endarterectomies. Biopsies were collected from the site of incision. This research was approved by the Sydney Local Health District Ethics Review Committee (Protocol No X14-0240), and informed consent was obtained and documented from all individuals. Deidentified fibroblast cultures were derived via the explant method [39], with dermal samples expanded on a gelatine-based 6-well plate and incubated with Hi-glucose DMEM containing 20% foetal bovine serum (FBS) media (with 1% NaPyruvate, penicillin and streptomycin, L-glutamine). When cells were confluent they were passaged to T75 flasks at 1:2. Reprogramming was initiated on passage 2 or 3.

### 2.2. Transdifferentiation

Patient derived adult fibroblasts, and human neonatal foreskin BJ fibroblasts (American Type Culture Collection (ATCC), Manassas, VA 20108), were seeded on 6-well gelatine coated plates in DMEM containing 10% FBS (with 1% NaPyruvate, penicillin and streptomycin, L-glutamine) overnight before transfection. Cells were transfected over 14 days, with mmRNA (RNACore, 6670 Bertner Avenue, Houston, Texas 77030) encoding ETV2, FLI1, GATA2, and KLF4 each day in the presence of Lipofectamine RNAiMAX (13778–075, L3484, Life Technologies Australia Pty Ltd., Scoresby, VIC 3179). In generating the mmRNA, we replaced uridine with pseudouridine and replaced cytosine with 5-methylcytosine, so as to reduce innate immune activation [40]. The cells were incubated in DMEM with 7.5% FBS and 7.5% knock-out serum replacement with 0.2 μg/ml B18R for the first 3 days, DMEM with 7.5% FBS and 10% knockout serum replacement with 0.2 μg/ml B18R, 50 ng/mL vascular endothelial growth factor, 20 ng/mL basic fibroblast growth factor, and 20 ng/mL BMP4 for days 4–7, and DMEM with 7.5% FBS and 10% knockout serum replacement with 0.2 μg/ml B18R, 50 ng/mL vascular endothelial growth factor, 20 ng/mL basic fibroblast growth factor, and 0.1mM 8-Bromo cAMP for days 8–14. After 14 days, cells were sorted for CD31+ using a CD31 Microbead kit and an OctoMACS Separator (130-091-935, Miltenyi Biotec Australia Pty. Ltd., Unit 16 A, 2 Eden Park Drive, Macquarie Park, NSW 2113, Australia). Sorted cells were cultured using EC growth medium EGM-2MV (CC3202, Lonza Group Ltd.) and a TFG-β inhibitor, SB341542 (10 μmol/L).

### 2.3. In vitro experiments

In vitro experiments were conducted comparing neonatal-derived iECs and patient- derived iECs. To examine for endothelial cell marker expression cells were fixed with PBS + 4% paraformaldehyde and stained with DAPI and anti-CD31 (560983, BD Biosciences, Becton, Dickinson and Company, 1 Becton Drive, Franklin Lakes, NJ 07417–1880, USA), with images obtained at 10X magnification. For the Matrigel tubulogenesis assay 1 × 104 cells/well were seeded on 6 wells of a 96-well plate coated with 35 μL of growth factor reduced Matrigel (Falcon, FAL356231, In Vitro Technologies, 7–9 Summit Rd., Noble Park North, VIC 3174, Australia) and incubated in EBM + 1% FBS for 24 hrs. Images were taken at 5 and 24 hours after seeding. To measure acetylated LDL uptake 5 × 104 cells/well were seeded on 3 wells of a 24-well plate in EGM2-MV. After 24 hours the cells were incubated with Dil-labeled acetylated-LDL (Low Density Lipoprotein from Human Plasma, Acetylated, Dil complex, L3484, Life Technologies Australia Pty Ltd., Scoresby, VIC 3179) for 4 hours and with UEA lectin (Lectin from Ulex europaeus, FITC conjugate, L9006, Sigma-Aldrich, 3050 Spruce St., St. Louis, MO 63103, USA) for 30 minutes prior to imaging. Migration towards VEGF was measured using a Boyden chamber assay; 1.1 × 104 cells/well were seeded in transwells (8 μm pore size, Corning Transwell, CLS 3421–22, Sigma-Aldrich, 3050 Spruce St., St. Louis, MO 63103, USA) in 100 μL EBM2 + 2% FBS media and these were inserted into 24 well plates. Experimental wells (3 wells) contained 600 μL EBM + 2% FBS and 10 ng/mL VEGF, while control wells (3 wells) contained EBM + 2% FBS alone, without VEGF. The cells were incubated for 24 hours. Transwell membranes were then washed, fixed with 70% ethanol, and stained with UEA lectin and DAPI. The total number of cells that had moved to the opposite side of the membranes were counted to assess migration. Cytokine secretion was measured in conditioned media for each cell line. Neonatal-derived iECs, patient-derived iECs, and human coronary artery endothelial cells (HCAECs) were seeded on 6 well culture plates (1 × 105 cells/well). After 24 hours, the media was changed to EBM2 + 2% FBS and the cells were incubated for 24 hours, under either hypoxic (1.2% O2) or normoxic (21% O2) conditions. The conditioned media was centrifuged at 2000 rpm for 10 min, before being stored at -80°C. The concentrations of a selection of angiogenic cytokines, VEGF, HGF, PlGF, SDF-1, FGF-1, FGF-2, and Leptin were measured using the Luminex polystyrene bead-based multiplex assay (Luminex High Performance Human Screening Assay, LXSAH-07, R&D Systems, Inc. Minneapolis, MN 55413, USA) as per manufacturer’s instructions. All samples were run in duplicate with three biological replicates for each cell line.

### 2.4. Hindlimb ischaemia model

In a murine model of PAD adapted from that developed by Niiyama et al. [41], Male NOD/SCID mice, aged 8 weeks, underwent unilateral femoral artery and vein ligation and removal to induce hindlimb ischaemia. Mice were randomly allocated to either the control group or one of the cell treatment groups, which included one neonatal-derived iEC treatment group, and three separate patient-derived iEC treatment groups. Immediately after surgical ligation and excision of the femoral vessels the mice received intramuscular injections of either 1 × 106 neonatal-derived iECs in EBM media, 1 × 106 patient-derived iECs in EBM media, or EBM media (n = 26 controls, 9 per treatment group). Cells were administered as two 25 μL injections into the adductor muscle adjacent to the site of the femoral vessels prior to removal. Perfusion in both hindlimbs for each mouse was measured using laser Doppler on days 0, 1, 2, 4, 6, 8, 10 and 14 post-surgery. During the surgical procedure and laser Doppler measurements the mice were anaesthetised via 1% isoflurane inhalation and positioned on a heat mat. Five repeat measurements were taken 5 minutes after anaesthetic induction, with the maximal perfusion measurement used in analysis.

At completion of the study gastrocnemius muscles were removed and snap frozen in OCT, with 5 μm sections of these samples stained with anti-CD31, anti-laminin (ab25644 and ab11576 respectively, Abcam plc. 330 Science Park, Cambridge CB4, UK) and anti-α smooth muscle actin (F3777, Sigma-Aldrich, 3050 Spruce St., St. Louis, MO 63103, USA) antibodies. Capillary density was measured by counting the number of capillaries per myocyte for each section.

Animal studies were approved by the Sydney Local Health District Animal Welfare Committee (Protocol Number 2015/020) and conducted in accordance with the National Health and Medical Research Centre (NHMRC) Guidelines for the care and use of animals for scientific purposes.

### 2.5. Statistical analysis

Data is presented here as mean ± SEM. Statistical analyses were performed using GraphPad Prism version 7.0b for Mac OS X, (GraphPad Software, La Jolla, California, USA). The Student’s t-test was employed for comparisons between 2 groups and ANOVA with Bonferroni post hoc testing for comparisons of multiple groups. Statistical significance is indicated at p < 0.05.

## 3. Results

### 3.1. Establishing patient-derived fibroblast cell lines

Dermal fibroblast cell lines established for four PAD patients were used for transdifferentiation and analysed in the in vitro studies. Throughout the remainder of this paper they are referred to as patients 1, 2, 3, and 4. Of these, patients 1, 2, and 3 were used for the *in vivo* hindlimb ischaemia study. The logistical constraint of slow cell growth precluded us from using the patient 4 cell line in the *in vivo* study. Patients had an array of cardiovascular risk factors including smoking, type 2 diabetes mellitus, hyperlipidaemia, and hypertension. Details associated with each patient are presented in Table 1. Values listed were obtained prior to surgery.

**Table 1:** Basic information regarding specific cardiovascular risk factors associated with patients who donated skin samples.

Patient	Age	Gender	Hyperlipidaemia	DM	Smoking	HTN	BMI
				(HbA1c)	(Pack years)		
1	64	Female	Yes	Yes (7.2%)	Yes (56)	Yes	27
2	64	Male	Yes	Yes (7.4%)	Yes (40)	Yes	-
3	70	Male	Yes	Yes (8.4%)	Yes (40)	Yes	18.9
4	88	Male	Yes	Yes (7.9%)	No	Yes	30.1

DM: diabetes mellitus type 2. HTN: hypertension.

### 3.2. Transdifferentiation of neonatal and patient derived fibroblasts to iECs

**Fig 1:**
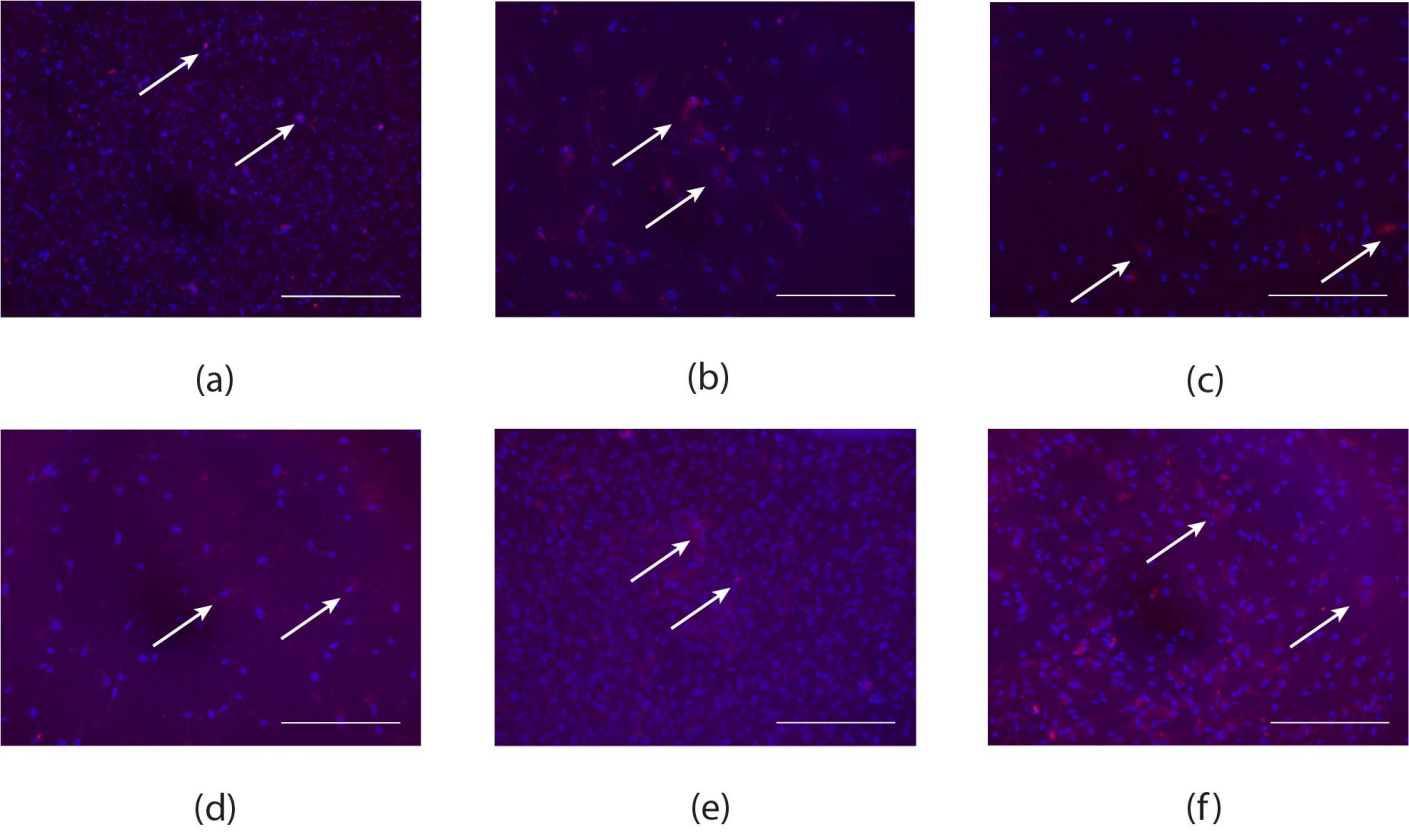
Representative immunofluorescence images of neonatal and patient-derived iECs. CD31 (red) immunofluorescence for (a) HCAECs, (b) neonatal-derived iECs; (c) Patient 1; (d) Patient 2; (e) Patient 3; (f) Patient 4, with cell nuclei stained purple using DAPI. Scale bars are 500 μm.

Upon completion of the transdifferentiation protocol CD31+ cells were successfully sorted from remaining cells. Neonatal and patient-derived iECs demonstrated CD31 expression via immunofluorescence (Fig 1).

### 3.3. Neonatal-derived and patient-derived iECs behaviour in vitro

**Fig 2:**
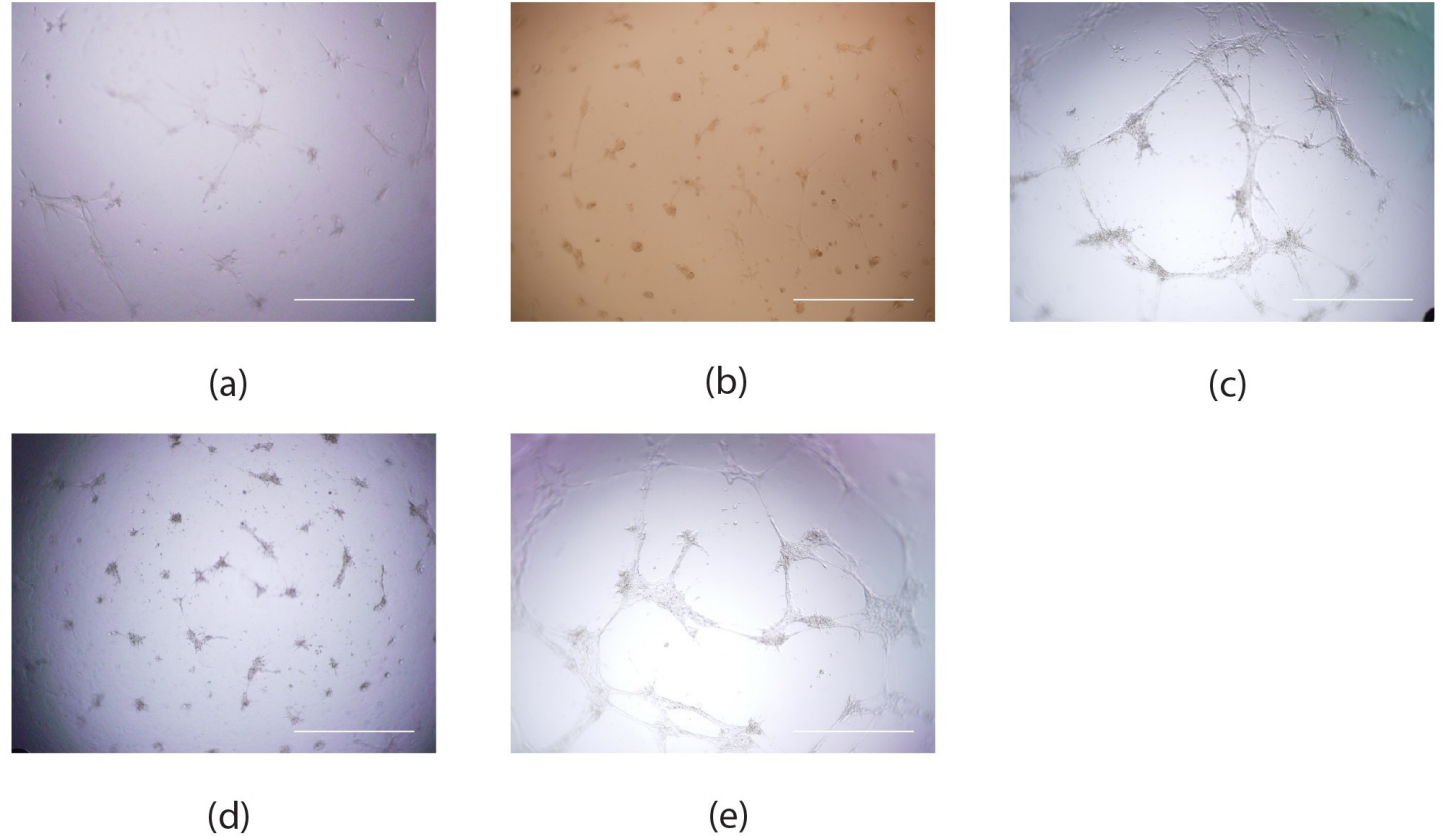
iEC tube formation after incubation on growth factor reduced Matrigel. (a) Neonatal-derived iECs; (b) Patient 1; (c) Patient 2; (d) Patient 3; (e) Patient 4, 24 hrs post seeding. Scale bars are 500 μm.

**Fig 3:**
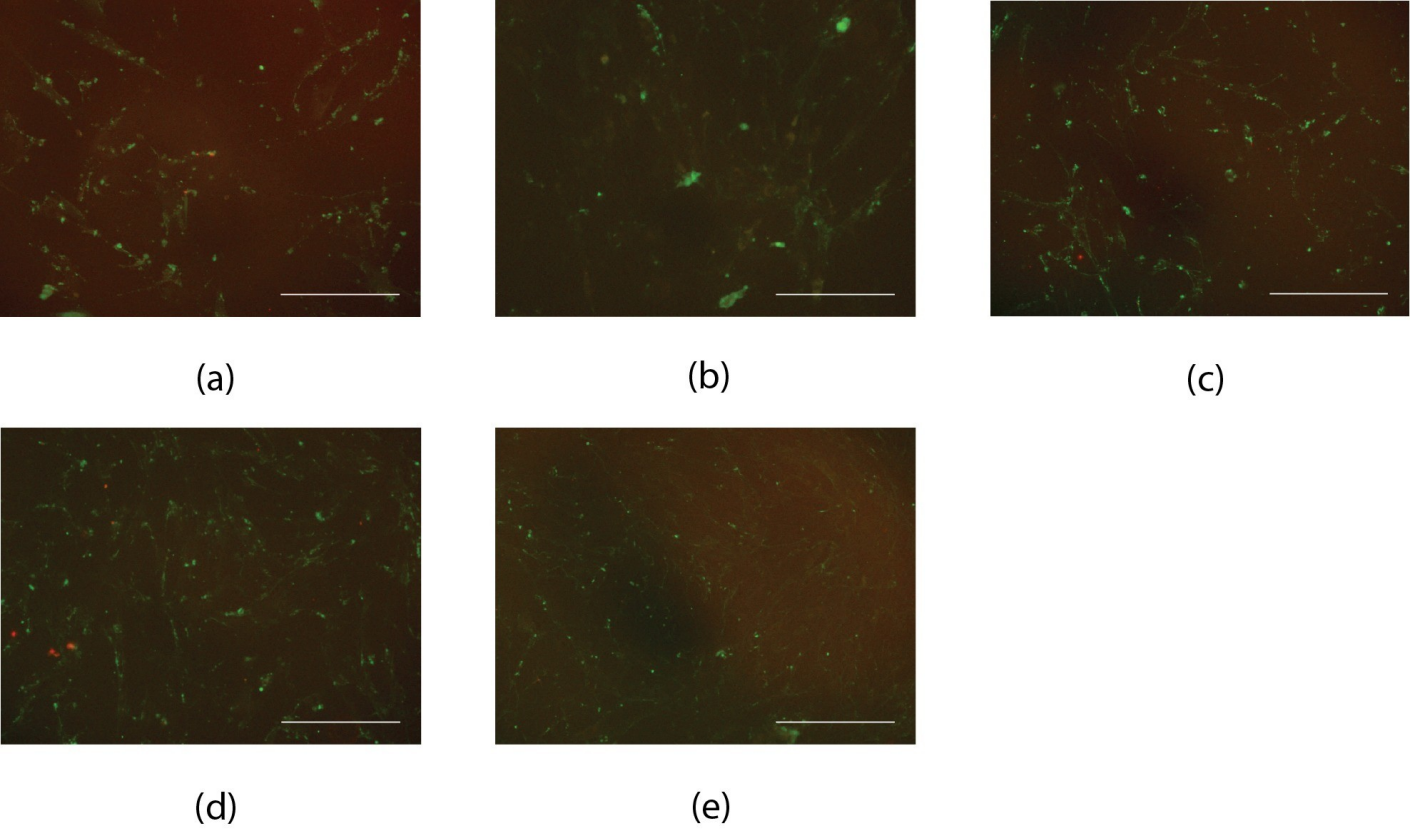
UEA lectin 1 binding and acetylated-LDL uptake for iECs. UEA lectin 1 (green) and acetylated-LDL (red) for (a) Neonatal-derived iECs; (b) Patient 1; (c) Patient 2; (d) Patient 3; (e) Patient 4. Scale bars are 500 μm.

Patient-derived iECs were compared against neonatal-derived iECs and HCAECs *in vitro*. In comparison to neonatal-derived iECs the capacity for branching was heterogeneous between patient-derived iEC lines (Fig 2); qualitatively similar tubulogenesis was displayed by patients 2 and 4, while patients 1 and 3 displayed limited tubule formation. Both neonatal and patient-derived iECs bound UEA lectin 1, but had limited uptake of acetylated-LDL (Fig 3). Non-significant positive trends in migration across Transwells in response to VEGF were found for each cell line (S1 Fig).

**Fig 4:**
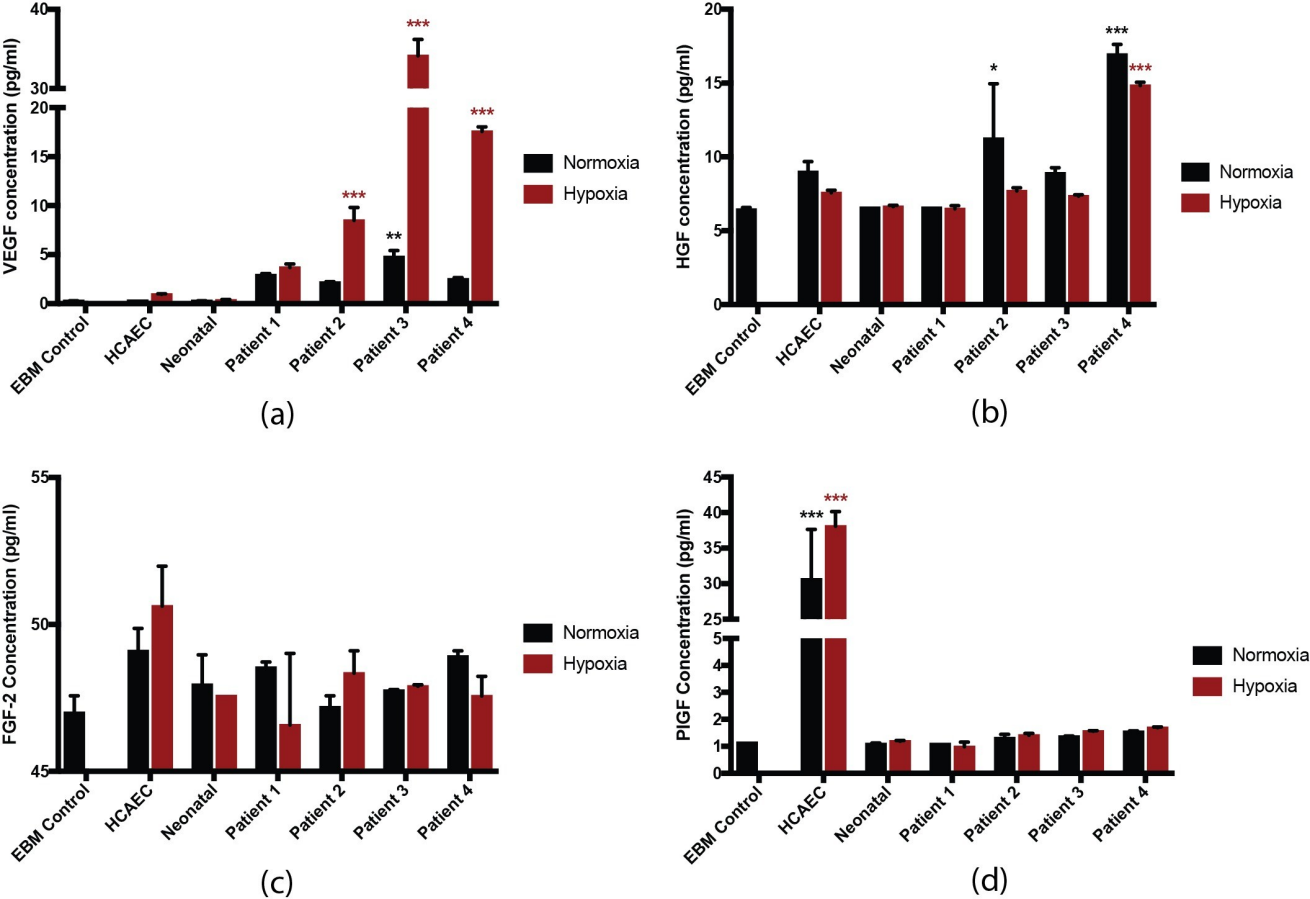
Levels of cytokine concentration detected in conditioned media for iECs. Cytokine concentration after culturing cells in normoxic and hypoxic conditions; (a) VEGF; (b) HGF; (c) FGF-2; (d) PlGF for HCAECs, neonatal-derived iECs and patient-derived iECs under normoxic (black) and hypoxic (red) conditions. Data were analysed by two-way ANOVA and are presented as mean ± SEM. (n = 3 per group, * p< 0.05, ** p< 0.01, *** p< 0.001 compared to neonatal normoxia; * p< 0.05, ** p< 0.01, *** p< 0.001 compared to neonatal hypoxia).

Using a multiplex assay, we tested conditioned media from HCAECs, neonatal- derived iECs, and each patient-derived iEC line for the secretion of angiogenic cytokines under normoxic and hypoxic conditions (Fig 4). HCAECs secreted detectable levels of VEGF, HGF, FGF-2, and PlGF. Neonatal-derived iECs demonstrated detectable secretion of VEGF and FGF-2. Patient-derived iECs demonstrated detectable secretion of VEGF, HGF, and FGF-2. In comparison to neonatal-derived iECs, patient 3 secreted significantly more VEGF in normoxic conditions (neonatal vs. patient 3, 0.3 pg/ml ± 0.1 vs. 4.77 pg/ml ± 1.1), and patients 2, 3, and 4 secreted significantly more VEGF in hypoxic conditions (neonatal vs. patient 2 vs patient 3 vs patient 4, 0.4 pg/ml ± 0.1 vs. 8.5 pg/ml ± 2.3 vs. 34.1 pg/ml ± 3.6 vs. 17.6 pg/ml ± 0.8). In comparison to neonatal-derived iECs, patient 2 and patient 4 secreted significantly more HGF in normoxic conditions (neonatal vs. patient 2 vs. patient 4, 6.6 pg/ml ± 0.1 vs. 11.2 pg/ml ± 6.4 vs. 16.9 pg/ml ± 1.2), and patient 4 secreted significantly more HGF in hypoxic conditions (neonatal vs. patient 4, 6.6 pg/ml ± 0.1 vs. 14.8 pg/ml ± 0.4). Secretion of FGF-1, SDF-1, and leptin was negligible for all cell lines.

### 3.4. Transplantation of neonatal-derived and patient-derived iECs enhances per- fusion recovery in ischaemic hindlimbs

**Fig 5:**
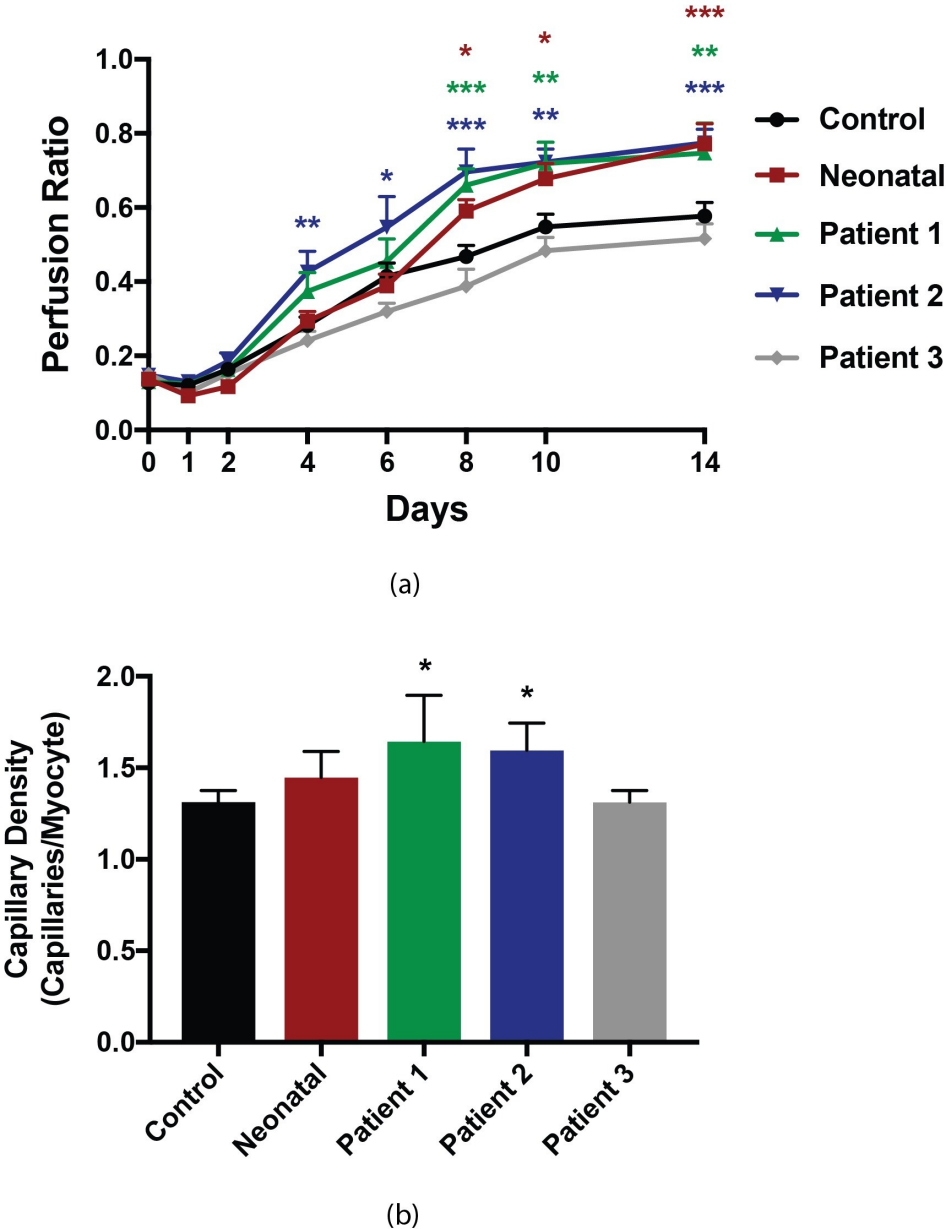
Blood perfusion recovery and capillary density in mice post hindlimb ischaemia surgery and treatment with iECs. (a) Blood perfusion recovery in ischaemic hindlimbs of mice treated with neonatal-derived iECs (red), patient-derived iECs from Patient 1 (green), Patient 2 (blue), and Patient 3 (grey), compared to controls (EBM) (black). Data were analysed by two-way repeated measures ANOVA and are presented as mean ± SEM (n = 23 for control group, n = 9 for iEC groups, * p< 0.05, ** p< 0.01, *** p< 0.001 compared to control. Asterix colour denotes associated treatment group). (b) Capillary density (capillaries/myocytes) for gastrocnemius muscle tissue from ischaemic hindlimbs collected at 14 days post-surgery for groups treated with neonatal-derived iECs (red), and patient-derived iECs for Patient 1 (green), Patient 2 (blue), and Patient 3 (grey), and control (EBM) (black). Data were analysed by one-way ANOVA with Bonferroni post hoc comparisons and are presented as mean ± SEM (n = 5–10 per group, * p< 0.05 compared to control).

Recovery in perfusion to ischaemic hindlimbs was superior in mice treated with neonatal-derived iECs in comparison those administered the vehicle control, EBM. The perfusion ratio was significantly enhanced in neonatal-derived iEC treatment group at days 8 (EBM vs. Neonatal-derived iECs; 0.47 ± 0.03 vs. 0.59 ± 0.03, p < 0.05), 10 (EBM vs. Neonatal-derived iECs; 0.55 ± 0.03 vs. 0.68 ± 0.04, p < 0.05), and 14 (EBM vs. Neonatal-derived iECs; 0.58 ± 0.04 vs. 0.77 ± 0.06, p < 0.001) (Fig 5) (Representative Doppler images in S2 Fig).

The recovery in perfusion to ischaemic hindlimbs observed in mice treated with patient-derived iECs was heterogenous, differing across patients. Recovery in perfusion was significantly increased in mice treated with patient-derived iECs generated from patient 1 and patient 2, but was not increased in those treated with iECs generated from patient 3 (Fig 5). Specifically, for patient specific treatment we found the following:

**Patient 1:** The perfusion ratio was significantly enhanced in the patient 1 iEC treatment group at days 8 (Controls vs. Patient 1 iECs; 0.47 ± 0.03 vs. 0.66 ± 0.04, p < 0.001), 10 (Controls vs. Patient 1 iECs; 0.55 ± 0.03 vs. 0.72 ± 0.06, p < 0.01), and 14 (Controls vs. Patient 1 iECs; 0.58 ± 0.04 vs. 0.75 ± 0.08, p < 0.01).

**Patient 2:** The perfusion ratio was significantly enhanced in the Patient 2 iEC treatment group at days 4 (Controls vs. Patient 2 iECs; 0.28 ± 0.02 vs. 0.43 ± 0.06, p < 0.01), 6 (Controls vs. Patient 2 iECs; 0.41 ± 0.04 vs. 0.55 ± 0.08, p < 0.05), 8 (Controls vs. Patient 2 iECs; 0.47 ± 0.03 vs. 0.70 ± 0.06, p < 0.001), 10 (Controls vs. Patient 2 iECs; 0.55 ± 0.04 vs. 0.72 ± 0.04, p < 0.01), and 14 (Controls vs. Patient 2 iECs; 0.58 ± 0.04 vs. 0.77 ± 0.04, p < 0.001).

**Patient 3:** The perfusion ratio was not significantly enhanced for any time point for the patient 3 iEC treatment group.

### 3.5. Enhanced perfusion associated with increased capillary density in iEC-treated mice

In comparison to the control group, mice treated with neonatal-derived iECs demonstrated a positive trend to increased capillary density, though this was non-significant (control vs. neonatal-derived iECs; 1.31 ± 0.20 vs. 1.45 ± 0.32, p> 0.05), and mice treated with patient-derived iECs from patients 1 and 2 demonstrated significantly increased capillary density (control vs. patient 1 vs. patient 2; 1.31 ± 0.20 vs. 1.64 ± 0.25, p< 0.05 vs. 1.60 ± 0.15, p< 0.05) (Fig 5). The patient 3 treatment group had capillary density similar to that of controls (control vs. patient 3; 1.31 ± 0.20 vs. 1.31 ± 0.19, p> 0.99).

## 4. Discussion

The potential benefit of cellular therapies produced via reprogramming is particularly high in conditions characterised by a loss of cell quantity and functionality. With cellular dysfunction being a major contributor to the pathophysiology of severe PAD the capacity to produce exogenous populations of cells via reprogramming techniques holds particular promise [12, 42–46]. Induced endothelial cells (iECs), a novel cell type produced via direct transdifferentiation of dermal fibroblasts to endothelial-like cells, represent a pro-angiogenic cell type that may be an effective cellular therapy in PAD [16, 22].

Use of viral vectors that integrate into somatic cell DNA limits the clinical viability of such cells due to safety concerns [16, 22]. Non-integrating vectors, such as mmRNA, do not alter the host genome and are more acceptable for the production of clinical grade cells [47]. Furthermore, while allogenic iECs may have future clinical applications, autologous patient-derived iECs, produced directly from a patient’s own dermal fibroblasts, represent a theoretically ideal cell type with respect to safety and potentially efficacy. In this proof of concept study, we aimed to determine whether PAD patient-derived fibroblasts could be transdifferentiated to iECs using non-integrating mmRNA and whether neonatal-derived and patient-derived iECs demonstrated comparable pro-angiogenic properties in vitro and in vivo.

We utilised an existing transdifferentiation protocol established for neonatal fibroblasts employing mmRNA encoding endothelial transcription factors ETV2, FLI1, GATA2, and KLF4. Using this protocol we demonstrated that patient-derived fibroblasts could be successfully transdifferentiated into iECs. Our findings demonstrate that age and other cardiovascular risk factors are not barriers to iEC generation.

We compared neonatal and patient-derived iEC behaviour in vitro. In comparison to neonatal-derived iECs the capacity for tubulogenesis was heterogeneous between patient-derived iEC lines. Previous studies have found variation in iEC phenotype, with iECs exhibiting effective tubulogenesis in one study [22], but not in the other [16]. Both neonatal and patient-derived iECs bound UEA 1 lectin, a marker of endothelial cells, and demonstrated limited uptake of acetylated-LDL. While both neonatal and patient-derived iECs demonstrated positive trends in their migration towards VEGF these were not statistically significant. These results were in line with previous results using iECs produced using lentiviral vectors [16].

Since it is hypothesised that iECs are proangiogenic due to paracrine secretion of pro-angiogenic cytokines [16], we assessed the levels of several such cytokines secreted by neonatal and patient-derived iECs in vitro in normoxic and hypoxic conditions. Cytokine levels were heterogeneous between patient-derived iEC lines and between normoxic and hypoxic conditions, suggesting functional differences between the cell lines. Interestingly, we found that the observed secretion levels for VEGF appeared to be higher for patient-derived iECs than neonatal-derived iECs in both normoxic and hypoxic conditions, with patients 2, 3, and 4 secreting significantly more VEGF than neonatal iECs. It is noted that PAD patients have increased circulating VEGF [48–52], consistent with these results.

The small number of patients included in the study precluded us from determining significant associations between patient specific characteristics and the heterogeneity we observed in the *in vitro* studies. Further studies are required to determine associations between age, glycaemic control, PAD severity, and smoking history and in vitro behaviour of patient-derived iECs.

We found that intramuscular injection of neonatal-derived iECs produced using mmRNA promote neo-angiogenesis in this model. This finding is consistent with existing results for iECs produced using lentiviral vectors [16]. Furthermore, in the first in vivo analysis of patient-derived iECs we found that patient-derived iECs have the capacity to promote neo-angiogenesis. Again, we noted heterogeneity in cell functionality across cell lines, with iECs derived from patients 1 and 2 showing significant improvement in perfusion, whilst iECs derived from patient 3 were not associated with any improvement. The observed improvements in perfusion post ischaemia were associated with increased capillary density in gastrocnemius muscles. Of note, the heterogeneity between recovery in perfusion for patient-derived iECs was conserved in the data relating to capillary density; iECs from patients 1 and 2 demonstrated increased capillary density while patient 3 was not associated with an improvement.

The above in vivo results were surprising, especially given the lack of response to iECs derived from patient 3, due to the high VEGF secretion in vitro associated with this cell line. We hypothesised that there must be some degree of uncoupling between the *in vitro* and *in vivo* behaviour of the iECs, and that the degree of VEGF expression observed in the assay was not an indicator of in vivo function. In fact, given that patient 3 had a particularly high level of VEGF expression, but demonstrated no significant increase in perfusion, and given that PAD patients have higher circulating VEGF levels, we hypothesised that perhaps very high levels of secreted VEGF may in fact be an indicator for cellular dysfunction. In addition, we also noted that the patient 3 iEC line demonstrated reduced capacity to form tubules in comparison to other patient-derived iEC lines. This in vitro behaviour may indicate a level of cellular dysfunction that is reflected in the poor response in vivo. Further work is required to analyse the robustness of the pro-angiogenic effect of patient-derived iECs. Larger studies are also required to determine whether there are statistically significant biomarkers that can be used to predict in vivo function of patient-derived iECs.

The heterogeneity between patient-derived cell lines in our in vitro and in vivo studies suggests that there may be patient specific differences between iECs that contribute to altered iEC phenotype. The observed heterogeneity between cell lines is of clinical significance, as this might result in some patients having minimal benefit from any future iEC derived therapy, whilst others have significant benefit. This would result in wasted resources with respect to using poorly performing iECs in specific patients, and furthermore, might limit the ability to detect a positive response for particular individuals in any future clinical trial.

The observed heterogeneity in the in vitro and in vivo results for different patient-derived iEC lines may be explained by differences in patient age and disease severity. As displayed in Table 1, patients’ ages ranged from 64 to 88. Advanced age and a greater degree of exposure to cardiovascular risk factors such as hyperlipidaemia, hyperglycaemia, and hypertension may be associated with a higher degree of cellular dysfunction and impaired pro-angiogenic capacity in vivo in iECs. Indeed, we noted that glycaemic control, as indicated by the HbA1c values prior to surgery, was worse for patient 3 in comparison to patients 1 and 2, and may represent a possible causal factor in the reduced perfusion response exhibited for the associated cell line. A more comprehensive study utilizing a larger patient cohort is required to investigate further the impact of individual risk factors on iEC behaviour. The possibility exists that there are clinical markers, differences in cellular function, or detectable genetic or epigenetic differences, that could predict which patient-derived iECs lines will be significantly pro-angiogenic.

### 4.1. Study limitations

We note that the in vitro comparison of iEC phenotype performed for this study, whilst investigating tubulogenesis, lacked a definitive in vitro assessment of angiogenesis such as via aortic ring or three-dimensional spheroid assays [53]. Important mechanistic differences differentiating effective and ineffective angiogenesis resulting from iECs could be elucidated by such assays, and would be a useful extension of this work. In addition, patient donors recruited for this study differed in gender, age, disease severity, as well as past medical history and current pharmacotherapy. The effects that patient specific factors have on iEC function have important implications for the clinical utility of this cell type. iECs derived from particular patient subgroups may be particularly effective or ineffective and this information is particularly important in developing iECs and other similar cell types for therapeutic uses in the future. Our study was not large enough to detect such differences, and a more extensive investigation of patient-derived iECs is warranted.

## 5. Conclusions

Neonatal fibroblasts and dermal fibroblasts derived from PAD patients can both be transdifferentiated to endothelial-like iECs using mmRNA. Though heterogeneity between patient-derived iECs was identified, both neonatal-derived and patient-derived iECs demonstrated qualitatively similar in vitro behaviour. Both neonatal-derived and patient-derived iECs demonstrated the capacity for pro-angiogenic behaviour in vivo in a hindlimb ischaemia mouse model of PAD, though the pro-angiogenic response was not consistent for all patient-derived iECs. These findings suggest that developing patient-derived iECs for therapeutic use may be feasible.

## Supporting information

S1 Fig iEC Transwell migration towards VEGF. Transwell migration towards VEGF relative to migration towards inert control. 100% represents level of migration observed towards inert control for each individual cell line. Data were analysed by two-way ANOVA and are presented as mean ± SEM.
(TIF)

S2 Fig Representative laser Doppler images for improvements in hindlimb perfusion. (a) control (EBM), (b) neonatal-derived iEC group across days 1, 2, 4, 6, 8, 10, 14, post-surgery.
(TIF)

S3 Fig Representative fluorescence microscopy images showing gastrocnemius muscle tissue from ischaemic hindlimbs collected at 14 days post-surgery, CD31 (red), Laminin (blue), smooth muscle actin (green); (a) control; (b) neonatal-derived iEC group; (c)-(e) patient-derived iECs: (c) patient 1; (d) patient 2; (e) patient 3. All scale bars are 200 μm.
(TIF)

S1 Data (XLSX)
