# Supplementary figures and images for: Induced endothelial cells from peripheral arterial disease patients and neonatal fibroblasts have comparable angiogenic properties
Source: PLoS One. 2021 Aug 10;16(8):e0255075. doi: 10.1371/journal.pone.0255075 (PMC8354451; doi:10.1371/journal.pone.0255075)

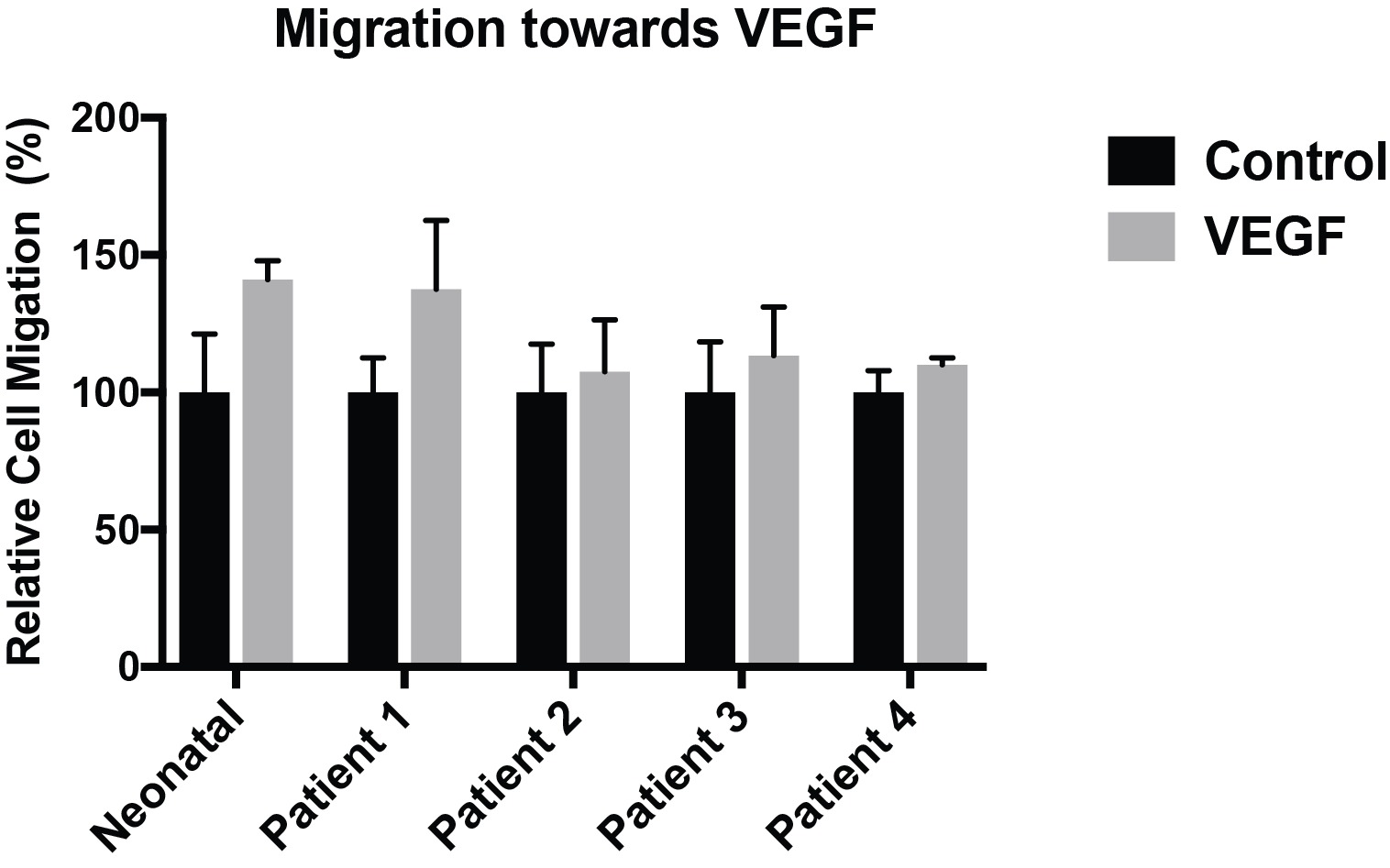

Supplement: S1 Fig — Transwell migration towards VEGF relative to migration towards inert control. 100% represents level of migration observed towards inert control for each individual cell line. Data were analysed by two-way ANOVA and are presented as mean ± SEM. (TIF) [file pone.0255075.s001.tif]

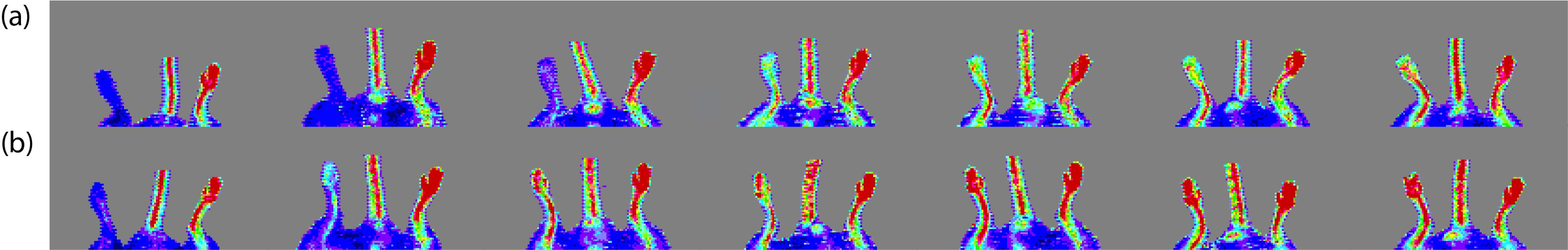

Supplement: S2 Fig — (a) control (EBM), (b) neonatal-derived iEC group across days 1, 2, 4, 6, 8, 10, 14, post-surgery. (TIF) [file pone.0255075.s002.tif]

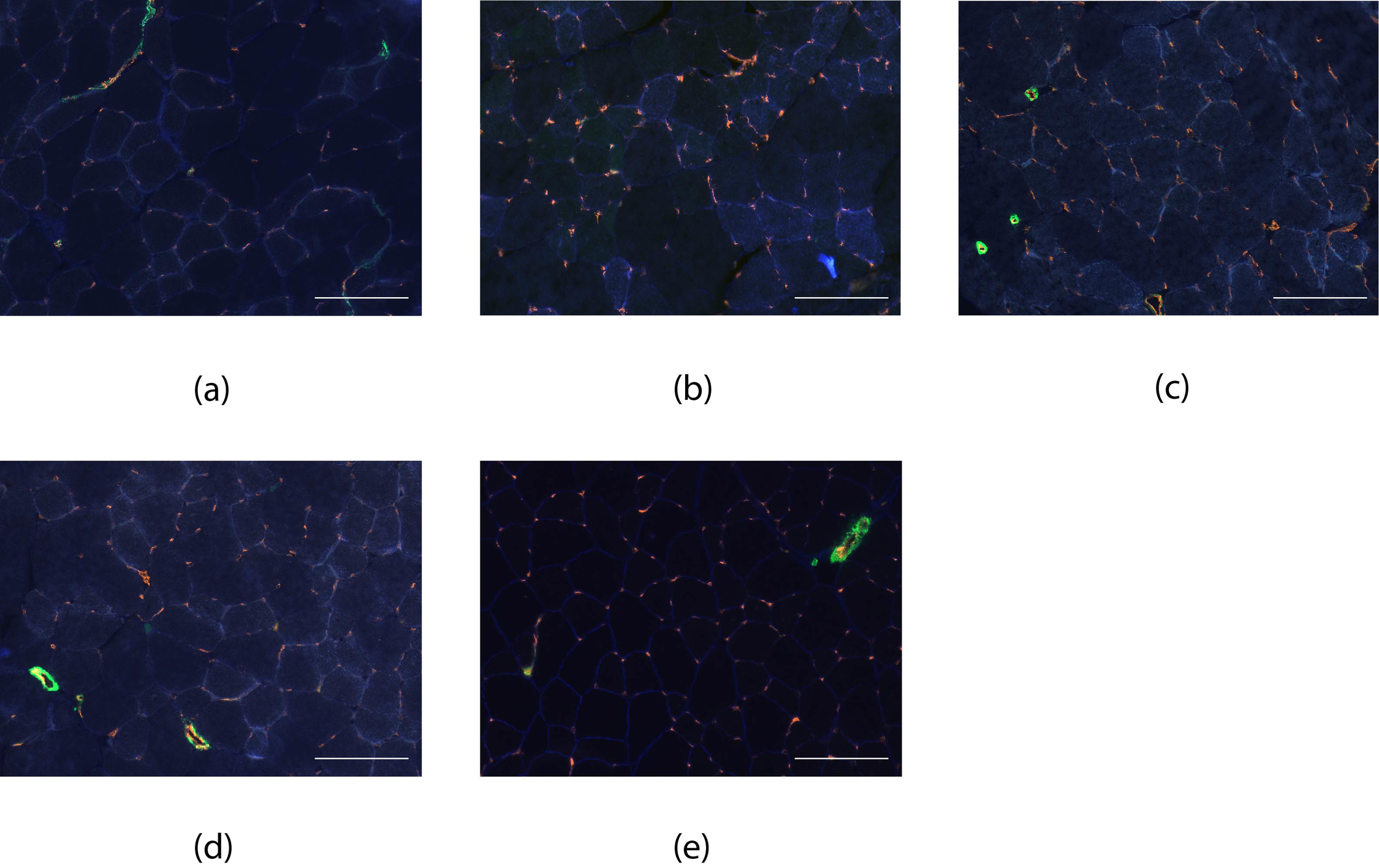

Supplement: S3 Fig — Representative fluorescence microscopy images showing gastrocnemius muscle tissue from ischaemic hindlimbs collected at 14 days post-surgery, CD31 (red), Laminin (blue), smooth muscle actin (green); (a) control; (b) neonatal-derived iEC group; (c)-(e) patient-derived iECs: (c) patient 1; (d) patient 2; (e) patient 3. All scale bars are 200 μm. (TIF) [file pone.0255075.s003.tif]
